# Communicating Food Risk-Benefit Assessments: Edible Insects as Red Meat Replacers

**DOI:** 10.3389/fnut.2021.749696

**Published:** 2021-12-16

**Authors:** Emilia Boehm, Dan Borzekowski, Ermolaos Ververis, Mark Lohmann, Gaby-Fleur Böl

**Affiliations:** ^1^Department Risk Communication, German Federal Institute for Risk Assessment, Berlin, Germany; ^2^European Food Safety Authority (EFSA), Parma, Italy; ^3^School of Medicine, National and Kapodistrian University of Athens (NKUA), Athens, Greece

**Keywords:** risk-benefit assessment (RBA), health communication, risk communication, food communication, food perception, edible insects, novel foods, red meat substitute

## Abstract

Risk-benefit Assessment (RBA) is an emerging methodology in the area of Food and Nutrition that offers a simultaneous evaluation of both risks and benefits linked to dietary choices. Communication of such research to consumers may present a challenge due to the dual nature of RBA. We present a case study of a communication strategy developed for the NovRBA-project. The NovRBA-project (Novel foods as red meat replacers—an insight using Risk Benefit Assessment methods) performed a risk-benefit assessment to evaluate the overall health impact of substituting red meat (beef) by a novel food (house cricket), considering the microbial, toxicological and nutritional characteristics of the respective dietary choices. A literature review of risk perceptions and acceptance of beef and insects as food formed the basis of the communication strategy for the study's results, drawing on environmental and emotional as well as health-related motivations to consume or avoid either food and considering the sociodemographic characteristics of likely consumers. Challenges and future directions for consumer protection organizations communicating findings of risk-benefit analyses on food safety are discussed.

## Introduction

In recent years, Risk-Benefit Assessment (RBA), a comparatively novel methodological approach for the field of food and nutrition, is gaining popularity (1, 2). RBA aims to assess both risks and benefits under a single methodological framework, providing a consolidated perspective on the impact that single foods, dietary options, or even whole diets may have on public health (3). To date, the most widely accepted practice in the scientific and regulatory food safety sector is the separate assessment of food risks and benefits (4). The more holistic approach allowed by RBA may present a valuable addition to existing standards and become more integrated into assessment practices in future (5).

Research interest in the field of food risk-benefit communication is relatively recent (6). For some risks, communication centered on risks only may be appropriate. However, in cases such as food consumption, communication considering both risks and benefits is preferable (7). For example, consuming certain fish species is linked to the risk of exposure to heavy metals and other contaminants, but the benefits, i.e., dietary intake of high-quality protein, omega-3 fatty acids, vitamins, and other essential nutrients, should also be considered (8, 9). Effective risk-benefit communication provides the information required to enable the consumer to make sound choices (10) and fosters and optimizes consumer protection (11). It may, therefore, allow for the prevention of undesirable environmental, economic and health-related outcomes (12). To allow consumers to make informed dietary choices and help risk managers and policy makers protect public health effectively, scientific risk-benefit assessments and their communication must reflect all aspects relevant to consumers' health.

There are many challenges to effective risk-benefit communication. Considering consumers' risk perception is crucial (13). Consequently, target audiences' risk perceptions must be understood before designing a communication strategy. Target audiences' varying sociodemographic characteristics, such as age, gender, educational level, and cultural background (14), or individual differences such as preferred channels for receiving information, must be explored and considered.

Frewer et al.'s (6) recent systematic literature review on risk-benefit communication regarding food highlighted the challenges associated with such communication and contributed to inform good practice. Reviewing 54 articles published between 1990 and 2011 and covering research conducted mainly in Europe and North America, the authors highlighted that reported communication sometimes related to risks and benefits (22 articles), but sometimes only to risks (29 articles) or only to benefits (three articles). The consideration of theoretical frameworks for communication was found in 20 of the 54 articles, with no single theoretical approach dominating. The impact of risk-benefit communication was infrequently assessed in terms of behaviors; instead, cognitive proxy measures such as intentions, perceptions, attitudes, or opinions were used, with risk perception being the most frequently studied dependent variable. This heterogeneity in communication focus (on both risks and benefits, or either individually), consideration and choice of theoretical frameworks, and outcome assessment variables demonstrates the complexity of the field and the challenges of developing, implementing, and assessing communication strategies regarding risks and benefits of food consumption. The authors note that a single solution suitable for risk-benefit communication is unlikely to emerge; instead, the specific needs of the target audience or identifiable sub-groups of particular vulnerability, namely their informational needs, existing behaviors, and habits, need to be considered when designing the structure and defining the relevance of communication content. Frewer et al. (6) identified three broad themes relevant to developing best practice in risk (benefit) communication: (1) the characteristics of the target population (2), the contents of the information communicated, and (3) the (perceived) characteristics of the information sources. Furthermore, they considered acute and chronic risk-benefit communication to require different focal points. While acute communication—e.g., in the context of a crisis—requires advances in communication processes, the timeframe of chronic risks allows for the consideration of more information on the topic (e.g., impact of the risk, who is affected etc.) and for messages to be tailored according to the respective risk-benefit perceptions and other variables. Chronic risk-benefit communication ought to be driven by audience requirements. In addition, Frewer et al. (6) suggest incorporating related behaviors of the audience and state that recommendations for changes in behavior should be concrete and actionable.

The eating habits of populations are ever-changing, which presents various challenges for risk assessments and risk-benefit communication. Reduced meat consumption, particularly of red meat, is among the current trends in Western societies; a development following a significant increase in red meat consumption over the last five decades in many regions across the globe (15). Consumers' interest to make dietary choices that promote their personal well-being as well as societal-level considerations regarding animal welfare, ethics, or environmental impact of food choices, may factor into current trends and should be considered for risk-benefit communication. Recent epidemiological studies have linked red meat consumption, especially of its processed products (16), to the development of chronic diseases such as diabetes type 2 (17, 18), colorectal cancer (19), and cardiovascular diseases (20). However, red meat also provides an array of macro- and micronutrients such as high-quality protein (21), iron, and vitamin B12 to the human diet (22, 23). Reducing red meat consumption could have a negative health impact through a reduced intake of these nutrients. Consequently, it is vital to consider the health implications of alternative diets reducing or eliminating red meat consumption. Various alternatives or substitutes for red meat are emerging. Legumes, insects, algae, and *in vitro* meat are among the foodstuffs trying to find their way into the plates of consumers in their capacity as protein sources (24, 25). When used as food ingredients, these alternative protein sources can mimic meat products such as sausage, burgers, and charcuterie.

Recent EFSA publications give positive indications regarding the safety assessment of insect-derived products (26–28), such as frozen and dried formulations of whole house crickets. These scientific opinions highlighted that the feed provided to insects may impact the contaminant profile of the insect-derived food since certain insect species have the ability to bioaccumulate heavy metals and other contaminants potentially present in the feed. The EFSA NDA Panel concluded that no safety concerns are raised in this respect as long as the administered feed complies with the applicable EU regulatory limits. EFSA also noted the presence of antinutrients, naturally occurring substances that can interfere with the absorption of nutrients in the body, in certain edible insect species. However, the levels of these compounds were comparable to those in other foodstuffs currently consumed. A separate concern are the allergic reactions consumption of edible insects may trigger in some consumers, including those already allergic to crustaceans, mites, and mollusks.

Within the above framework, the NovRBA project (Novel foods as red meat replacers—an insight using Risk Benefit Assessment methods) aims to estimate the overall health impact of replacing red meat with novel protein sources, using insects, and Acheta domesticus (house cricket) in particular, as a case study. Around 2000 insect species are reportedly consumed by various cultures worldwide (29), but in the majority of the western world, entomophagy is not considered a common practice. However, insect species are increasingly found in food markets, mainly as ingredients in a vast range of products (30). In fact, the first insect species to be used as food has been recently approved in the European Union (31), following a positive safety assessment by the European Food Safety Authority (32). *Acheta domesticus* has been reported to have an elevated commercial potential in the food market of the European Union (33, 34), accompanied by a plethora of published data on its compositional profile.

We present here the generation of an evidence-based communication strategy for the NovRBA project's results to target audiences in order to support healthy, informed consumption choices. Literature reviews of risk perceptions and acceptance of red meat and insects as food were conducted. Although the environmental impact of food production may also be considered for communication (35), the literature review focused on human health-related perceptions due to the NovRBA project's overall focus. The literature reviews aimed to provide an information basis for the project' communication strategy by providing information about potential cognitive and cultural barriers to or facilitators of consumption as well as likely sociodemographic and regional differences in target groups.

## Materials and Methods

### Search Parameters and Search Strings

Literature reviews on the public perception of risk and related constructs, including acceptance, in relation to (1) edible insects and (2) red meat were carried out, aiming to map the pan-European population's risk perceptions, state of knowledge, and information requirements on these topics. The electronic bibliographic database “Scopus” was searched through 3rd March 2020, using the following search strings:

“(accept^*^ OR perc^*^) AND (“edible insects” OR entomophagy) in [TITLE-ABS-KEYS]”

“(accept^*^ OR perc^*^) AND (“red meat” OR beef OR pork) in [TITLE-ABS-KEYS]”

No restrictions regarding publication language or year were applied. Additional studies or publications relevant to the topic that were not identified through the literature search but known to the authors, such as a representative population survey carried out in Germany (36) by the German Federal Institute for Risk Assessment (BfR), were added to the list of relevant publications.

### Abstract Screening

To select relevant publications, a researcher scanned abstracts to evaluate their relevance. All references for which a link to risk perception and related theoretical constructions as distinct from other perceptions of insects as food or red meat respectively were included for the final literature review. The abstracts of potential references were scanned according to the above criteria (pan-European relevance, risk perception, population's state of knowledge, and information requirements), and relevant publications were included in the final literature review. Inclusion or exclusion of publications was performed irrespective of the study design.

## Results of the Literature Reviews

### Results of the Literature Review: Summary of Findings on Risk Perception Relating to Insects as Food

The literature search yielded 150 unique references; 33 publications were identified as most relevant from the article abstracts and included in the literature review (see Table 1). Key information extracted from each publication in order to inform the design of the communication strategy are summarized below.

**Table 1:** Overview of the publications selected as relevant to risk perception relating to edible insects in the literature review.

**References**	**Study type (sample size)**	**Key findings/topics**
Baker et al. (37)	Online experiment in the United States of America (*N*_1_ = 221; *N*_2_ = 200; *N*_3_ = 201)	Visual or descriptive information had an impact on risk perceptions and purchase intent.
Batat and Peter (38)	Literature review	Development of a conceptual framework identifying key factors related to the acceptance and adoption of insect-based foods in Western food cultures.
Caparros Megido et al. (39)	Online survey and experiment in Belgium (*N* = 79)	Insect tasting sessions decreased *food neophobia*.
De Boer et al. (40)	Online survey in the Netherlands (*N* = 1,083)	The Dutch population showed a positive attitude toward a change to a diet with more environmentally friendly proteins, with the exception of insects.
DeFoliart (41)	Literature review	Comparison of the perception and consumption of insects as traditional foods with the Western attitude toward edible insects.
Gere et al. (42)	Online survey in Hungary (*N* = 400)	*Food neophobia* was the main barrier to insect consumption.
Gmuer et al. (43)	Online survey in Switzerland (*N* = 428)	Disgust/uneasiness, inertia/dissatisfaction and positive emotional evaluations predicted *willingness to eat insects*.
Hamerman (44)	Online survey in the United States of America (*N* = 179)	Different aspects of disgust reduced *willingness to eat insects*.
Hartmann et al. (45)	Online survey in Germany (*N* = 502) and China (*N* = 443)	Chinese participants rated insect-based foods more favorably than German participants. They also indicated greater willingness to eat the tested food products.
Hartmann and Siegrist (46)	Experiment in Switzerland (*N* = 104)	Exposure to processed insect products can increase consumers' willingness to consume unprocessed insects.
Hartmann and Siegrist (47)	Literature review	Europeans‘willingness to consume insects was considered very low. Higher willingness was associated with male gender.
Jensen and Lieberoth (48)	Online survey and experiment in Denmark (*N* = 189)	Perceived social norms predicted the *willingness to eat insects*.
Kim et al. (49)	Literature review	Entomophagy increases worldwide, despite its unfamiliarity to the consumers influenced by Western eating habits.
Lensvelt and Steenbekkers (50)	Online survey and experiment in the Netherlands (*N* = 134) and Australia (*N* = 75)	Information and providing the opportunity to try insect food positively influenced the attitude toward entomophagy.
Lombardi et al. (51)	Experiment in Italy (*N* = 200)	*Food neophobia* and beliefs and attitudes toward insects negatively affected the *willingness to pay* for insect-based products.
Mancini et al. (52)	Literature review	Acceptability of edible insects in European countries was the topic of very few publications.
Manhartseder (53)	Online survey in Austria (*N* = 164)	There was no effect of type of information on the *willingness to pay* for insect-based food products.
Meixner (54)	Online survey in Austria, Germany and Switzerland (*N* = 620)	The consumption of insects was not perceived as particularly risky.
Menozzi et al. (55)	Online survey in Italy (*N* = 231)	Beliefs in the positive effects on health and the environment positively impacted intention to consume insects-based foods. Disgust, incompatibility with local food culture, and lack of availability negatively impacted the intention.
Meyer-Rochow and Hakko (56)	Experiment in Italy (*N* = 26)	Insects were not easy to identify by taste alone.
Orsi et al. (57)	Online survey in Germany (*N* = 393)	Low willingness to try insects. Disgust and *food neophobia* were identified as one of the main barriers. Few participants perceived insects as unsafe.
Pambo et al. (58)	Field experiment in Kenya (*N* = 432)	Providing product information on insect-based products affected sensory evaluation of the products' sensory attributes.
Piha et al. (59)	Online survey in Finland, Sweden, Germany and the Czech Republic (*N* = 887)	Distinct types of knowledge and *food neophobia* affected willingness to buy, mediated by general attitudes.
Ruby et al. (60)	Online survey in the United States of America (*N* = 179) and India (*N* = 220)	Perceived benefits of eating insects were related to nutrition and environmental sustainability, and the most common risks related to risk of disease and illness.
Schäfer et al. (36)	Telephone survey in Germany (*N* = 1,000)	Insects as food and feed are known to a majority of the German population and they are rather seen as beneficial than as risky. The main reasons against insects as food are disgust and unfamiliarity.
Schosler et al. (61)	Online survey in the Netherlands (*N* = 1,083)	Meal formats, product familiarity, cooking skills, preferences for plant-based foods and motivational orientations toward food had in impact on the intention to prepare the presented meals at home.
Tan et al. (62)	Experiment in the Netherlands (*N* = 103)	Food appropriateness, but not the experienced sensory-liking, *food neophobia* or gender predicted willingness to eat unusual food among Dutch beef consumers.
Tan et al. (63)	Experiment in the Netherlands (*N* = 100)	Taste expectations were more negative when a food had never been tested before. Low willingness to eat was linked to food appropriateness more than the food's actual taste.
Tan et al. (64)	Experiment (*N* = 135) and online survey (*N* = 79) in the Netherlands	Appropriate product context improved expected sensory-liking and willingness to buy mealworm products.
Van Huis (65)	Literature review	Focusing on ecological and economical aspects, the paper provides insights into the rearing of insects.
van Huis (66)	Conference proceeding	Discussion of research pathways to make insects a viable sector in food and agriculture.
Verbeke (67)	Online survey (*N* = 368) in Belgium	*Food neophobia* made the largest contribution to consumers' readiness to adopt insect substitution.
Verneau et al. (68)	Experiment in Denmark (*N* = 141) and Italy (*N* = 141)	Communication was effective on intention and behavior regarding the willingness to eat insect-based food.

Lensvelt and Steenbekkers (50) identified factors influencing consumers' acceptance of entomophagy based on previous literature, differentiating between factors relating to the product (including price and quality, benefits, risks, naturalness, and fit with consumer needs and convenience), social trust and norms (e.g., trusted information sources), and psychological factors (including attitude and culture; prior attitude such as feelings of fear, dislike, and indifference, food neophobia, culture, and social influence). To enhance consumer acceptance, they recommend the approval of products by government and consumer organizations, promoting and raising awareness of product benefits through marketing (focusing on nutritional and environmental benefits), giving information about the manufacturing process, highlighting product fit with consumer needs and convenience, providing product information through trusted sources (e.g., consumer organizations), and improving public perception (through education, cook books, exhibitions, articles, etc.), possibly by using processed rather than unprocessed forms of insect-based food or highlighting resemblance to the familiar reference product. An online survey among Dutch (*n* = 134) and Australian (*n* = 75) consumers (*n* = 209) provided insight into attitudes toward entomophagy. Respondents were selected using a social network; accordingly, the sample is not representative of the respective populations. Neither Dutch nor Australian participants perceived eating insects as a risk (M_Dutch_ = 3.36; _MAus_ = 3.15; response scale 1–5). The risk perception item's wording is not reported. Participants were further asked to explain the risks they associated with eating insects in an open question. Out of 167 participants who answered this question, 50 did not associate any risk with eating insects, 35 mentioned that insects could carry diseases, bacteria or infections and 27 participants responded that people might get sick after eating insects or think that eating insects is too “scary.” Participants did not consider entomophagy beneficial to themselves personally, but perceived possible environmental benefits linked to insect consumption. Participants considered naturalness of products important, and associated insects with natural food. Participants believed information to be trustworthy when provided by scientific researchers, well-known relatives, the government, and persons using the product, but not when provided by food producers or famous persons. Dutch participants trusted information provided by consumer organizations more than Australian participants. Participants indicated they would be more likely to eat insects if mixed into a dish rather than consumed individually, with higher agreement by Australian compared to Dutch participants. Both were self-reportedly more likely to consume processed forms of insects. In a tasting component, 138 Australian participants were given the choice to consume insect-based food products after receiving information about factors related to the product, social norms and trust, physiological factors, or no information; attitudes toward insects were measured at the start and end of the experiment. The type of information offered did not impact the degree to which participants liked insect products' taste, nor their attitudes toward entomophagy at the end of the study. Attitudes of participants who chose to consume insects were neutral before and somewhat positive after the tasting, indicating beneficial effects of tasting experience on entomophagy attitudes. Participants with past experience of consumption were more likely to consume insect products.

Orsi et al. (57) report findings from a web-based survey with 393 German participants. The survey was distributed *via* social media, web forums, and contact lists; consequently, the survey sample was not representative of the population. The sample comprised 51% female and 49% male participants, reflecting the distribution in the German population. Participants were aged 13–82 years (M_Age_ = 36 years). Risk perception relating to edible insects was measured using a scale containing four items: (1) “Insects are unhygienic and transmit diseases,” (2) “Eating insects is risky,” (3) “Eating insects poses a risk to human health,” and (4) “Insects are not suitable for human consumption.” Answers could be given on a 5-point Likert scale. The paper does not report statistical data in relation to the risk perception scale, though stating that few participants perceived insects as unsafe.

Jensen and Lieberoth (48) report findings from an online survey with 189 Danish undergraduate students. Participants were young adults (*M* = 21.7, *SD* = 0.25) and predominantly female (159 women); consequently, the findings are not representative of the population. In an experimental component, participants' willingness to eat insects was later tested in an actual food tasting scenario. The Pathogen Disgust Scale was used to measure disgust toward various sources of pathogens, including questions on rotten food, others' bodily fluids, animal feces, and insects, using items of the format “Please rate how disgusting you find…” Descriptive statistics were not provided for the individual items (such as the question on insects) but reported at the scale level (overall Pathogen Disgust Score). Insect eating disgust was measured as a separate construct using three items: (1) “Eating food with insects is disgusting,” (2) “I am afraid of getting sick from eating food with insects,” and (3) “I get nauseous at the thought of eating food with insects.” Participants rated the degree to which they agreed with each statement on a 7-point Likert scale (1 = “Completely disagree;” 7 = “Completely agree”). An overall insect eating disgust score was created by averaging these three items, which were highly correlated (*r* = 0.44 to 0.70, *p* = 0.001) with good internal consistency (α = 0.78), indicating that participants tended to express similar degrees of agreement for all three items. Descriptive statistics for the individual items were not reported but participants' overall insect eating disgust scores were neither high nor low on average (*M* = 3.58, *SD* = 0.25, response scale = 1–7). Insect eating disgust was not correlated with general Pathogen Disgust despite the inclusion of an insect disgust item in the Pathogen Disgust Scale used, indicating distinct cognitive processes for considering general disgust of insects and other pathogen-related situations and objects vs. disgust of insects in particular in the context of consumption. This suggests that disgust at eating insects may not be linked to an emotional (disgust) response at a potential pathogen but rather be driven by other factors. Perceived Infectability (participants' belief to be particularly susceptible to catch infections of various sorts) was also not correlated with insect eating disgust, further supporting the interpretation of these results that fear of contamination/disease/illness may not be a driving factor of disgust of insect consumption.

In Jensen and Lieberoth's laboratory food tasting component, 153 participants (81%) consumed insect-based products. Only 101 (53%) participants had previously indicated they would definitely or probably try mealworms if offered, showing that self-reported willingness to consume insects (intention to consume) may not correspond fully with actual consumption behavior. Accordingly, research using self-reported willingness to consume as a proxy measure for consumption behavior should be interpreted with some caution, as their findings may not generalize to actual consumption behavior. Behavioral intention for eating insect-based food (willingness to eat) with visible mealworms was notably lower (*M* = 2.48, *SD* = 1.44, response scale = 1–7) than participants' willingness to eat insect-based products with invisible mealworms (*M* = 4.47, *SD* = 1.73, response scale = 1–7). This indicates that acceptability of consuming insect-based foods may be higher, at least with respect to behavioral intention, for processed as opposed to unprocessed foods. Specifically, the visibility of insects in the food seems to be a barrier, possibly due to the animal reminder evoking associations which inhibit willingness to consume the food.

Insect eating disgust significantly predicted insect tasting behavior, while general pathogen disgust and perceived infectability did not. This suggests that whether an individual is easily or strongly disgusted by pathogen-related objects or situations or whether they consider themselves highly susceptible to infection are not factors that drive the decision to consume insects. Instead, the disgust toward eating insects is closely linked to insect consumption behavior and both appear to be separate from other fears of infection and contamination.

Another significant predictor of insect tasting behavior was perceived social norm. This was measured on a 100-point scale (*M* = 68.67, *SD* = 18.57) using two items assessing participants' belief about how many others had consumed insect-based foods at the tasting session. Those consuming insects themselves tended to believe greater numbers of others were consuming insects. This could indicate that social acceptability and perceived norm are crucial factors in (initial) insect consumption behavior, while individuals' disgust toward pathogens or perceived infection susceptibility do not contribute notably to this decision.

Insect tasting (behavior) was decreased by food neophobia (*r* = −0.29, *p* < 0.001) and insect eating disgust (*r* = −0.36, *p* < 0.001), but increased by past experience of eating insects (*r* = 0.17, *p* < 0.05) and willingness to eat mealworms (behavioral intention; *r* = 0.44, *p* < 0.001). This suggests that weariness of consuming novel foods in general (food neophobia) and disgust of eating insects in particular are barriers toward insect consumption, while past experience and positive behavioral intention favor insect consumption. The significant, but moderate correlation between willingness to consume mealworms and insect consumption highlights the need to distinguish between behavioral intention and behavior, as they cannot be fully equated.

Schäfer et al. (36) conducted a telephone survey, recruiting a sample representative of the German population of *N* = 1,000. Respondents were aged 14 and over. The survey focused on the perception of insects as food and feed.

Almost 14% of respondents had consumed insects in the past, with 5.5% having consumed insects on a single occasion, 5.2% on two to five occasions, and 3.2% on more than five occasions. Consumption was more frequent among individuals with higher educational attainments, those living in urban rather than country regions, and male as opposed to female respondents. Respondents aged 18–29 and 30–39 reported past consumption with higher frequency than other age groups, with those aged 14–17 or 65+ reporting the least past consumption. Of those with past consumption experience (*N* = 139), future consumption was rated as very probable by 34.3% and as rather probable by 36.2%, while 20.2% rated it as rather improbable and 8.2% as very improbable (1.2% didn't know). Past consumption experience occurred in Germany in 44% of cases, with 63.6% abroad.

Risk perception was operationalized in two ways. Firstly, a “health factor” was measured using the item “How healthy do you consider the consumption of insects?” Five categories and a no-response option were offered as response choices: healthy, fairly healthy, neither healthy nor unhealthy, fairly unhealthy, unhealthy; don't know. The consumption of insects was considered healthy or fairly healthy by 46.7% of participants, while only 15% considered it to be unhealthy or fairly unhealthy, 36.3% considered it neither healthy nor unhealthy and 2% indicated that they did not know.

Secondly, risk perception was measured directly by asking participants whether they believed that the consumption of insects induces health risks for humans or animals. Responses were given as “Yes,” “No,” or “Don‘t know.” A majority of 62.5% participants indicated their belief that consumption of insects does not induce health risks, while 26.1 and 19.7% of respondents believed consumption to pose a health risk to humans and animals, respectively. Respondents were less concerned about the use of insects as feed than as food, with 63.3% of respondents overall in favor of using insects as feed (27.1% were overall not in favor of this use) but only 46.6% overall in favor of using insects as food (47.6% were overall not in favor of this use). Insects as food were seen more favorably among those living in cities than those living in the countryside. Differences were observed between different age groups, with 18–29-year-olds most favorable and 14–17-year-olds and those aged 65+ least favorable. Individuals with lower levels of educational attainment also indicated less favorable views of insects as food than individuals with a high school or university degree.

Respondents believing a health risk to be linked to consumption were asked to specify possible health risks in an open question. The *transfer or outbreak of diseases* was listed by 54.5% of respondents, followed by *toxins* (26%), and *allergies/intolerances* (16.8%).

When asked to list possible reasons for consuming insects (*N* = 1,000), high protein content was the reason most frequently listed (33.7%), followed by providing additional sources of nutrition for the world population (17.1%), nutrition/vitamin content (14.2%), low costs and effort of husbandry (13.1%), high availability (12.3%), providing an alternative to presently available food (7.3%) or feed options (6.9%), sustainability of insect husbandry (5.2%), pleasant taste (4.6%), establishment as food in other cultures (2%), curiosity or interest in trying the novel consumption experience (1.8%), a view of insects as a normal food and edible animal species similar to others (1.7%), a healthy consumption choice (1.6%), or easy to prepare (0.6%), with 1.5% listing other reasons and 14.9% indicating they could not think of any possible reasons for consuming insects.

Asked to list possible reasons for not consuming insects, disgust was listed by far the most frequently (45.7%), followed by concerns about hygiene and insects as carriers of diseases (14.9%), the lack of familiarity with insects as a food (13.4%), animal abuse concerns and lack of trust in suitable husbandry practices (11.8%), interference in natural ecosystems/threat of extinction or loss of species diversity (11.2%), avoidance of animal-based products or meat-free diets (4.4%), digestibility (4.0%), concerns over taste (3.4%), the availability of sufficient other foods (2.7%), concerns over preparation (1.3%), poisonous quality (0.9%), difficult husbandry (0.8%), and contaminants (0.7%), with 1.1% listing other reasons and 11.4% indicating they could not think of any possible reasons not to consume insects.

Lombardi et al. (51) conducted an experiment with 200 students at an Italian university which investigated the role of information and carriers (specific insect-containing products—pasta, cookies, and chocolate bars) on the willingness to pay for insect-based food. Different carriers generated different results in terms of willingness to pay for conventional and insect-based versions of the products. Food Neophobia and Beliefs and Attitudes toward insects negatively affected the willingness to pay. In the adapted version of the beliefs and attitudes toward insects scale used, several items relating to risk perception were assessed. Participants' belief that eating insects would increase risk of infectious disease was relatively low (*M* = 2.52, *SD* = 1.504, Range = 1–7). Relating to insects generally rather than as a food, participants' belief that insects carry harmful microbes (*M* = 3.09, *SD* = 1.642, Range = 1–7) or contain harmful toxins (*M* = 2.83, *SD* = 1.368, Range = 1–7) was also rather low than high (low ratings indicated low belief). Participants believed that insects are highly nutritious (*M* = 4.75, *SD* = 1.613, Range = 1–7), while fewer participants believed that humans eating insects is not natural (*M* = 2.97, *SD* = 1.842, Range = 1–7). A group comparison contrasted the impact of individual vs. community health benefit information on willingness to pay, showing a positive effect of either type of information with a slightly higher impact of individual benefits information.

Meixner (54) conducted a web-based survey with participants from Austria, Germany and Switzerland (*n* = 620). To measure risk perception, a scale consisting of four Items was used: (1) “Insects are unhygienic and dirty,” (2) “The consumption of insects poses a hazard to human health,” (3) “Insects are not suitable for human consumption,” and (4) “The consumption of insects is fraught with risk.” The consumption of insects was not perceived as particularly risky (*M* = 23.82, response scale 0–100).

In the context of a master's thesis Manhartseder (53), risk perception relating to insects as food was measured in Austrian participants using a web-based survey. Participants rated their agreement with five statements relating to insects (1 = strongly disagree to 7 = strongly agree). Respondents were aged 16–65 (*n* = 164, *M* = 34.03, *SD* = 12.71) and 59.6 % of the sample were female; the sample was not population representative.

Participants' relatively high agreement with “They are not presently part of our diets” (*n* = 164, *M* = 6.01, *SD* = 1.76) indicated insects were viewed as a novel food. “Insect products are identical to any other new, unfamiliar foodstuff introduced to the market” received much lower agreement (*n* = 144, *M* = 3.81, *SD* = 2.06), suggesting that insect-based foods may be considered a special case of novel food. If true, this could indicate that cognitive processes involved in the perception, acceptance and decision to consume relating to novel foods in general might not apply to insect-based foods and should thus not be generalized unless based on information about consumers' willingness to consume insects in particular. “Insects are not edible” received low agreement (*n* = 161, *M* = 2.86, *SD* = 1.92), indicating a tendency to view insects as edible rather than not. “Insects are horrible” was positively agreed with (*n* = 159, *M* = 5.34, *SD* = 1.98), but “Insects are hazardous and can transmit diseases” was barely agreed with (*n* = 135, *M* = 3.55, *SD* = 2.01). This indicates that factors other than attributions of hazard and disease contributed to the perception of insects as “horrible.” Agreement with “Insects are not the solution to our environmental problems with respect to meat consumption” (*n* = 149, *M* = 5.52, *SD* = 1.96, Range = 1–7) may present a tentative indication that barriers to insect consumption in this sample outweighed any environmental motivations that might have incentivised consumption.

A systematic literature review (47) on the perception and acceptance of insects as food focuses on European willingness to consume insects and its predictors, and strategies to increase acceptance of edible insects. Europeans' willingness to consume insects is considered very low, with survey data indicating willingness to consume insects as a meat substitute in only 19% of the population. Higher willingness to consume was associated with male gender, but no other sociodemographic factors were identified as impacting the willingness to consume insects as food. No associations with food contamination, health risks or primitive diets were found in people's perception.

A study conducted in the Netherlands (63) explored the sensory perceptions of three unusual novel foods claimed to be present in beef burger patties. Participants consumed burger samples with different novel ingredient proportions. Taste expectations were more negative when a food had never been tested before. Consumption experience was determined mainly by the food's properties. Low willingness to eat was linked to food appropriateness more than the food's actual taste.

Verneau et al. (68) demonstrated that communication choices could positively impact willingness to consume insects in 282 university students (Denmark: *n* = 141; 65 Females, *M* = 23.35, *SD* = 3.40; Italy: *n* = 141; 74 females, *M* = 23.87, *SD* = 4.25). Highlighting (1) social benefits or (2) individual benefits of introducing insects' proteins into human diet (experimental groups) or (3) information irrelevant to insects (control group) affected participants' behavioral intentions and consumption behaviors. Highlighting consumption benefits impacted behavioral intention positively; this effect carried over to consumption behavior. The impact of information on social benefits on behavioral intention was more long-lasting than information on individual benefits. Increased familiarity and male gender positively affected intention to consume. Negative implicit attitudes toward insects were identified as a barrier to consumption, though this did not impede the positive impact of health information. Higher levels of consumption intention and behavior were found in the Danish than the Italian sample, indicating potential effects of food culture: the well-established Italian cuisine may reduce cultural openness to novel foods. Gere et al. (42) conducted a study to understand the readiness of Hungarian consumers to adopt insects. *Food neophobia* was identified as the main barrier to insect consumption.

A literature review on factors affecting entomophagy acceptance and adoption in Western food cultures (38) provides a conceptual framework identifying key factors related to acceptance and adoption of insects and insects-based foods.

Tan et al. (62) explored the contribution of sensory-liking and food appropriateness to the willingness to eat unusual food among Dutch beef consumers. Food appropriateness, but not the experienced sensory-liking nor individual traits like *food neophobia* or *gender*, predicted willingness to eat unusual foods.

Verbeke (67) investigated Belgian consumers' readiness to adopt insects as a meat substitute. Gender, age, familiarity, food neophobia, convenience and environmental food choice motives, as well as meat-related attitudes and future meat consumption were identified as significant predictors. Food neophobia made the largest contribution to consumers' readiness to adopt insect substitution.

De Boer et al. (40) examined the relationship between motivational differences in food orientation and the choice of snacks made from “environmentally friendly proteins,” including insects. The results indicate that there is potential for a dietary shift toward “environmentally friendly proteins” among Dutch consumers, but only 4% of participants chose insect-based snacks. A hybrid meat and meat-substitute snack was chosen most frequently, though consumers with high involvement in food, taste and reflection orientation and high levels of education and an urban background were more likely to choose plant-based alternative sources of protein (in this study, lentils, and seaweed).

Kim et al. (49) reviewed current trends related to insects as food resources among consumers, industry, and academia, revealing a steady increase in entomophagy worldwide. van Huis (66) overview of edible insects as an alternative protein source for human food and animal feed discusses research pathways to make insects a viable sector in food and agriculture. Mancini et al.'s (52) literature review on European consumers' readiness to adopt insects as food noted that acceptability of edible insects in European countries was the topic of very few publications.

An entomophagy perception survey and hedonic test study among Belgian participants (39) concluded that insect tasting sessions are important to decrease *food neophobia* and that insect integration into Western food culture will involve a transitional phase with minced or powdered insects incorporated into ready-to-eat preparations.

An experiment with 26 Italian students (56) found that insects were not easy to identify by taste alone. The authors suggest that consumer acceptance may be facilitated when insects are processed into flour or pastes and reduced when packaging displays insect images.

A literature review (65) on edible insects focusing on ecological and economical aspects provides insights into the rearing of insects and highlights the development of scientific research and its focus on ecological concerns and economical implementation in this field. Another literature review (41) contrasts the perception and consumption of insects as traditional foods with the Western attitude toward edible insects.

A web-based survey with 428 Swiss participants (43) investigated willingness to eat insects. Participants made fewer positive evaluations and more negative evaluations of snacks containing insects than snacks not containing insects based on their images. Willingness to eat insects was predicted by disgust/uneasiness, inertia/dissatisfaction and positive emotional evaluations. The authors suggest the marketing of snacks containing processed insect ingredients.

A study with 179 undergraduate students at a US university (44) investigated factors influencing willingness to attend an insect tasting. Different aspects of disgust (animal reminder and core disgust) reduced willingness to taste insects. Following a priming to reflect on cooking processes, willingness to attend the tasting was less reduced in those with low sensitivity to animal remind disgust.

An experiment with 231 young, Italian adults (55) measured behavioral intention and action of eating novel food products containing insect flour in the following month. Beliefs in the positive effects on health and the environment positively impacted attitudes and intention to consume. Disgust, incompatibility with local food culture, and lack of products in the supermarket were barriers to consumption intentions.

A field experiment in Kenya with a community sample of 432 participants (58) investigated how consumers evaluated sensory attributes of buns blended with cricket flour. The effects of information on the evaluation, personal involvement and emotions were also tested. Providing product information affected sensory evaluation of the products' sensory attributes.

In a sample of 887 participants from Finland, Sweden, Germany and the Czech Republic, Piha et al. (59) compared how consumer knowledge influences willingness to buy insect food products in central and northern Europe. Distinct types of knowledge and food neophobia affected willingness to buy mainly indirectly, mediated by general attitudes. Subjective and objective knowledge predicted willingness to pay in northern but not central Europe, food neophobia in central more than in northern Europe. Product experience was a predictor in both regions.

An online survey (61) on Dutch consumer practices related to meat, meat substitution and meat reduction demonstrated the impact of meal formats, product familiarity, cooking skills, preferences for plant-based foods and motivational orientations toward food on the intention to prepare the presented meals at home (*N* = 1,083).

An experiment (46) in which 104 Swiss participants consumed traditional tortilla chips or cricket flour chips suggested that acceptance of insect consumption in unprocessed form might be increased after having consumed insect-based chips.

Studying willingness to consume insects in American and Indian samples, Ruby et al. (60) showed perceived benefits of eating insects were related to nutrition and environmental sustainability, and the most common risks related to risk of disease and illness (*N* = 399).

In a combined experimental and web-based survey study using a Dutch sample (64), consumers evaluated appropriate and inappropriate mealworm products along with original mealworm-free products (*N* = 135 willing tasters for the sensory study; *N* = 79 unwilling tasters for the online study). Appropriate product context improved expected sensory-liking and willingness to buy mealworm products. Mealworm products were expected and experienced to taste different from original mealworm-free products, but taste preferences were similar to the original.

Baker et al. (37) investigated the role of contextual information on insect consumption, specifically the impacts of images and descriptions on risk perceptions and purchase intent (three experiments; *N*_1_ = 221; *N*_2_ = 200; *N*_3_ = 201). The results suggest that, in restaurant settings, risk perception may be increased when food images contain insects in animal form, though the difference was observed only in combination with a vague, not an explicit text description. In retail settings, packaging image (containing an insect or not) impacted risk perception; text description (vague or explicit) did so only when combined with an image. Explicit text descriptions increased risk perceptions. This study's results could indicate that visual or descriptive information elicit disgust responses, which may then sensitize consumers to potential consumption risks.

In a web-based survey (45) of German (*n* = 502; *M*_Age_ = 44.3, *SD*_Age_ = 14.2, Range_Age_ = 20–69; Proportion male = 0.48) and Chinese (*n* = 443; *M*_Age_ = 44.2, *SD*_Age_ = 13.1, Range_Age_ = 20–69; Proportion male = 0.49) samples, Chinese participants rated insect-based foods more favorably and indicated greater willingness to eat the tested food products than German participants, who also reported higher willingness to eat processed insect-based foods compared to the unprocessed foods.

### Key Themes Extracted From the Literature Review: Risk Perception Relating to Insects as Food

#### Disgust and Animal Reminder

Disgust toward eating insects is a barrier to consumption. There is some evidence that this disgust does not relate to general pathogen disgust of perceived susceptibility to infection (48), which would suggest that fear of disease/contamination/illness is not the origin of disgust toward eating insects. Animal reminder disgust, one of the three main disgust domains alongside core and contamination disgusts (69), was highlighted as a barrier to willingness to taste insects (44). Participants less sensitive to animal reminder sensitivity were better able to overcome their disgust and consider that cooking transforms the insect-derived ingredients, classifying them as food and thus reducing the insects' “animalness.” Animal reminder sensitivity was found to be greater in women than men, which could factor into gender differences in insect consumption readiness. The impact of animal reminder disgust may be reduced by processed or unprocessed state of the food and may explain some of the reduced willingness to consume unprocessed compared to processed state insect-based food. Animal reminder disgust may also contribute to the increased risk perception evoked by insect-containing food images and explicit text descriptions (37).

#### Familiarity (First-Time vs. Repeat Consumption) and Food Neophobia

Insect consumption or proxy measures (e.g., attitudes, behavioral intention) are higher among individuals with past consumption experience in some settings (48, 50, 68) but not others, for example when food contained whole animals (57). The context of past consumption (a one-off novelty experience vs. cultural habits) may influence this relationship. The presence or absence of additional barriers or facilitating factors, such as processed vs. unprocessed state of insect-based food, may confound effects of familiarity.

Individuals high in food neophobia, a general weariness of consuming novel foods, are less likely to consume insects as a novel food (42, 48) and less willing to pay for (51, 59, 62) or consume (67) insect-containing products. The stronger relationship between food neophobia and willingness to pay for insect-containing food in central compared to northern Europe (59) indicates that this relationship is likely impacted by other barriers and facilitating factors.

#### State (Processed vs. Unprocessed)

Several studies indicate that consumption of insect-based foods is more acceptable, in particular for individuals with low willingness to consume insects (45) and those who have not tried insects before (57). Willingness to consume insects in unprocessed form may be increased in individuals who have consumed insects in the past (48), suggesting that introducing individuals to insect-based foods in processed form may increase the likelihood they will consume insects in unprocessed form in the future. Therefore, familiarity effects are likely to interact with the state of insect-based foods in their impact on individuals' willingness to eat insect-based foods and should be considered in future studies relating to the state of insect-based foods and their impact on willingness to eat. Unprocessed forms of insect-based food may also reduce animal reminder disgust and constitute an effective way to reduce this barrier to consumption as well as the barrier of novelty.

#### Contextual Information (Text, Images, and Source)

Any effects of contextual information on insect consumption behavior and attitudes are likely to represent a complex interplay of factors.

The degree of trust in the sources providing information may impact their effect, with some indication that scientific researchers, well-known relatives, the government, and persons using insect-based food products being trusted while food producers and famous persons may not be (50). There may however be cultural differences in the respective ratings of different informational sources; for example, consumer organizations may be more trusted by Dutch than Australian participants (50).

Information given about products seems to have inconsistent effects on attitudes and behavior. Some evidence suggests that the choice to consume insects, willingness to pay, or consumption acceptance are not influenced by information received (50, 53, 54). Other findings indicate that information about social of individual benefits of introducing insects' proteins into human diet positively impacted intention to consume, with longer-lasting effects from information highlighting social benefits (68). In contrast, highlighting individual or community health benefits of insect consumption was found to impact willingness to pay positively (51), but with greater impacts from highlighting individual benefits. Underlying differences in the effect of individual vs. collective benefit perception may impact differently on investment choices (e.g., by measuring willingness to pay) than on other constructs related to insect consumption, such as intention to consume. Explicit product descriptions highlighting the animal content on the other hand may reduce purchase intent, though such effects may interact with the presence of visual stimuli (37).

The visibility of insects as whole animal and visual animal reminders (e.g., images evoking thoughts of the live animals) may constitute a barrier to consumption (37, 48). These findings are consistent with animal reminder disgust as a barrier to consumption and the facilitating effects of processed as opposed to unprocessed forms of insect-based foods. However, there is some evidence than the effects of insect images interact with contextual text information given (37). It is likely that information (visual or text) that evoke thought of the live animal elicit animal reminder disgust and/or increase risk perception, thus reducing consumption behaviors or proxy measures thereof. However, this effect may not be detectable when animal reminder disgust is evoked by other contextual sources (e.g., the impact of visual information may be detectable only when animal reminder disgust is not already evoked by text descriptions).

The inconsistency in findings regarding contextual information seem to indicate a complex interplay of factors each associated with a comparatively low effect size. Large enough studies and samples to identify small effects, investigate the interactions between various contextual information factors, and account for the impact of other factors on consumption attitudes, intentions, and behaviors, would be needed to reliably investigate the relative roles of various contextual informational factors and their interactions.

#### Cultural Differences, Social Norms, and Contexts

Insect consumption and related cognitions and intentions are affected by cultural and social context and norms. Individuals are more likely to consume insects when they believe many of those surrounding them to do so as well (48). This indicates that humans do not inherently fear or avoid insect consumption (e.g., based on biological instincts), but rather that avoidance is a result of not having experienced the food as safe to consume based on others' (social norm) and the individual's own (familiarity) experience. Cultural differences such as the lower willingness to consume insect-based foods in a German compared to a Chinese sample (45) support this notion. Addressing social factors and perception could therefore be an effective strategy in promoting insect consumption. For example, consumption of insects or sharing of positive past consumption experiences by trusted persons in individuals' social circles (e.g., friends or family) might improve the perception of insect-based food consumption as a social norm and standard, rather than an exception. Positive consumption experiences among individuals low in barriers such as disgust would therefore be expected to positively influence the views and consumption behaviors of their social circles.

#### Sociodemographic Characteristics

There was a high degree of variability in the sociodemographic criteria of the samples of the studies reviewed. Given the likely impact of cultural influences on insect consumption perceptions, intentions, and behaviors, cross-cultural comparison studies conducted using samples representative of the respective populations would be required to reliably identify the role of sociodemographic criteria in diverse contexts. In particular, it is difficult to investigate the role of age on insect consumption behaviors and attitudes, partly because many of the above studies were conducted in disproportionately young populations due to the use of student samples or web-based methodologies. The identification of male gender as a facilitating factor for willingness to consume insects in a systematic literature review (47) is a promising indicator that women may be less likely to consume insects than men. Such differences may be linked to increased animal reminder sensitivity sometimes shown in women compared to men (44). Regional differences in insect consumption behaviors, intentions, and attitudes have been identified in various studies (45, 59, 68) and indicate that social and cultural contexts notably impact individuals' attitudes and behaviors.

### Results of the Literature Review: Risk Perception Relating to Red Meat

The literature search yielded 332 unique references; 12 publications were identified as most relevant from the article abstracts and included in the literature review (see Table 2). Key information extracted from each publication in order to inform the design of the communication strategy are summarized below.

**Table 2:** Overview of the publications selected as relevant to risk perception relating to red meat in the literature review.

**References**	**Study type (sample size)**	**Key findings**
Angulo and Gil (70)	Telephone survey in Spain (*N* = 650)	Beef was considered as one of the least safe products. Higher beef risk perception was linked to lower confidence in food safety in general.
Branscheid et al. (71)	Experiment in Germany (*N* = 200)	Investigation of consumers' sensory ratings in relation to sampled beef and lamb. Risk perception about beef was not measured.
Branscheid (72)	Literature review	Discussion of the quality of beef including the proportion of muscle to fatty tissue. Risk perception about beef was not measured.
Dwan and Miles (73)	Online survey in the United Kingdom (*N* = 167)	Participants more ready to accept the link to cancer had more negative attitudes toward red meat (including perceived health risks and benefits).
Gaspar et al. (74)	Online experiment in the United Kingdom, Belgium and Portugal (*N* = 174)	Individuals low in information avoidance had less positive attitudes and higher perceived knowledge relating to red meat.
Gutkowska et al. (75)	Survey in Poland (*N* = 1,004)	The third most frequently stated reason for beef consumption was “It is healthy,” with “Due to health-related reasons” the second least frequently stated.
Hornibrook et al. (76)	Survey and interview in Ireland (*N* = 687)	Risk perception was measured only in relation to purchasing choices, with food safety being the most important factor in purchasing choices. Avoidance of physical risks was rated most important.
Schlup and Brunner (77)^a^	Questionnaire in Switzerland (*N* = 378)	Perceived healthiness of meat was a negative predictor of participants willingness to consume insects.
Schroeder et al. (78)	Survey in Canada, the United States of America, Japan and Mexico (*N* = 4,005)	Beef was considered very safe or somewhat safe by the majority of respondents in Canada and the US. Most respondents in Japan and Mexico considered beef either mostly safe or neither safe nor unsafe.
Van Wezemael et al. (79)	Focus groups in France, Germany, the United Kingdom and Spain (*N*_groups_ = 8, *N*_participants_ = 65)	Beef was generally perceived as healthful, but the participants expected positive as well as negative effects of beef consumption on their health.
Van Wezemael et al. (79)	Focus groups in France, Germany, the United Kingdom and Spain (*N*_groups_ = 8, *N*_participants_ = 65)	Participants experienced difficulties in the assessment of the safety of beef and beef products.
Van Wezemael et al. (80)	Online survey in France, Germany, Poland, Spain and the United Kingdom (*N* = 2,520)	Consumers were overwhelmingly confident about purchased beef and beef product.

a *The main results of this study align with the NovRBA project's design to evaluate health outcomes of scenarios in which red meat is substituted with edible insects. Consideration of edible insects as a potentially healthier alternative to red meat products is therefore an integral part of the communication strategy; consequently, the findings of this study are not discussed independently below*.

A population representative telephone survey in the Spanish population (70) showed participants (*n* = 650) considered beef as one of the least safe products (*M* = 2.61, *SD* = 1.43, response scale = 1–5; higher scores indicating greater safety perception). Out of the 18 food products rated by participants, the items “beef” and “imported food” were joined for the second least safe food product, with only “ready-to-eat meals” perceived as less safe and thus the least safe item. Pork (*M* = 3.66, *SD* = 1.05, Range = 1–5) was perceived as notably safer, ranking 7th least safe out of the 18 food products. Standard deviation of safety perception ratings was greatest for beef, indicating great variability among consumers' risk perceptions. According to the authors, this pattern may result from differential impact of food scares on less experienced beef consumers who rely on mass media as an information source, which amplifies a negative perception of food safety in contrast with more experienced consumers who rely on other sources of information such as public authorities and whose food safety perceptions are less impacted by food scares and mass media. Angulo and Gil (70) propose an economic framework for the relationship between food and beef safety perceptions, socioeconomic variables, beef safety incidents, risk retrievers and purchasing likelihood. Applying this framework, higher beef risk perception was linked to lower confidence in food safety in general as well as lower frequency of purchasing beef and lower per person consumption levels. The authors argue that more experienced beef consumers may draw information about the product from wider sources of information, resulting in greater trust in food safety information provided by public authorities. The price of beef products was also linked to beef risk perception, with higher prices linked to higher perceived risk. Though the relationships between beef risk perception and socioeconomic characteristics were not investigated in this study, general confidence in food safety was higher in individuals (1) with higher education levels, (2) less influenced by mass media in their purchasing decisions, and (3) paying more attention to food labels and more confident in the information included on them.

Dwan and Miles (73) report the findings of an experimental study performed on a community sample of 167 community-dwelling participants in the UK. The impact of health information about the link between red meat and cancer on participants' belief that red meat can cause cancer was measured. Attitudes toward red meat (including ratings of agreement relating to the nutritional properties and health benefits of red meat) were also assessed, but sample means were not reported in this study. Participants more ready to accept the link to cancer risk had more negative attitudes toward red meat (including, but not limited to, perceived health risks and benefits), evaluated the information provided more favorably, were lower in ambiguity aversion and meat consumption. However, because sample means of participants' attitudes toward red meat were not reported and acceptance of the risk associated with red meat consumption was only measured after exposure to specific health information, no inferences about population risk should be drawn.

Gaspar et al. (74) investigated changes in attitudes and perceived risk knowledge relating to red meat following exposure to health information and the role of information avoidance tendencies in British, Belgian and Portuguese participants (*N* = 174). Perceived knowledge and attitudes toward red meat were measured before as well as after exposure to health information. Overall sample means were not reported. Individuals low in information avoidance had less positive attitudes (*M* = 4.85, *SD* = 1.38, Range = 1–7) and higher perceived knowledge (*M* = 4.32, *SD* = 1.06, Range = 1–7) relating to red meat than individuals high in information avoidance (Attitudes: *M* = 5.51, *SD* = 1.19, Range = 1–7; Knowledge: *M* = 4.10, *SD* = 0.67, Range = 1–7). Exposure to health risk information about red meat led to a decrease in positive attitudes and an increase in perceived knowledge in both high and low information avoiders. This indicates that both groups' risk perception can be influenced by information, though it may constitute a challenge to engage high information avoiders in the risk communication process which would expose them to such information.

A survey of 1,004 Polish participants (75) assessed beef consumption habits and related motives and beliefs. The third most frequently stated reason for beef consumption was “It is healthy” (39% of respondents), with “Due to health-related reasons” the second least frequently stated (10% of respondents). The percentage of respondents between 26 and 40 years of age indicating health benefits as the reason for their beef consumption was higher than in other age groups. The relative importance of different factors or sources of information for consumers' decision to buy beef are reported, with press articles (7%), advertising (9%), labeling information (10%), seller's recommendation (10%), and exposition in the store (12%) receiving the lowest number of top ratings, while the desire to prepare a specific dish (44%), respondents' own expertise/wont (34%), medical advice (24%) and opinions of others, e.g., family, friends (19%) received the highest number of top ratings. However, medical advice also received the second highest number of lowest ratings (29%), indicating that some individuals are highly influenced by medical advice, while others are very little influenced by this factor.

Hornibrook et al. (76) conducted a survey on 687 Irish consumers who buy pre-packed beef in supermarkets to assess their perceived risk and risk reduction strategies. Risk perception was assessed only in relation to purchasing choices, with food safety being the most important factor in purchasing choices. When comparing the relative importance of risks relating to financial, time, psychosocial, physical and performance losses, the avoidance of physical risks (food poisoning and consuming meat from an animal with BSE) was rated most important. Individuals with children rated the avoidance of physical risks as more important than individuals without children. Participants also rate the usefulness of various information sources in relation to beef. Consumer-dominated sources of information (e.g., past experience, recommendations from family and friends, quality assurance symbols) were rated the most useful (*M* = 4.00, *SD* = 0.83, Range = 1–5; *M* = 3.94, *SD* = 0.85, Range = 1–5; *M* = 3.83, *SD* = 0.85, Range = 1–5, respectively). Information from consumer organisations (*M* = 2.78, *SD* = 0.83, Range = 1–5), the department of health (*M* = 2.89, *SD* = 0.75, Range = 1–5), and the Food Safety Authority of Ireland (*M* = 3.06, *SD* = 0.75, Range = 1–5) were rated as far less useful in contrast. Gender and age differences in the usefulness ratings of different sources of information were also detected; for example, older participants rated past experience as more useful.

Van Wezemael et al. (79) report findings from 8 focus group studies with 7–9 participants each conducted in Germany, Spain, France and the United Kingdom. All participants were beef eaters. Consumer perceptions of beef are reported on the basis of a qualitative analysis. The majority of participants considered beef as healthful and of positive nutritious value (specifically with respect to iron and proteins) resulting in positive health effects. High trust was expressed in food regulation. Few consumers worried about potential negative health effects of beef; carcinogenic, cardiovascular and other long-term effects were mentioned in this context, as well as possible disease transfer from animals to humans and obesity. Health risks were perceived to depend on consumption factors such as amount, type, preparation method and the presence of harmful residues in the beef. Participants associated unhealthfulness with cheap or low quality beef, beef with hormones or additives, packaged or canned beef or further processed beef products, beef sold or processed in unhygienic conditions, offals, expired beef, ready meals and BSE/food crises.

Van Wezemael et al. (80) collected data from 2,520 regular beef consumers (504 each from France, Germany, Poland, Spain and the United Kingdom) in an online survey. The study focused on assessing consumer acceptance of beef product and processing safety interventions, though various measures relating to safety perceptions are also reported. Results showed consumers to be overwhelmingly confident about purchased beef and beef product, though means and standard deviations were not reported.

Schroeder et al. (78) report results from an international survey with 4,005 participants from Canada, the US, Japan and Mexico aiming to better understand consumer perceptions relating to beef in target markets for Canadian beef. Beef safety perception was measured, showing that beef was considered very safe or somewhat safe by the majority of respondents in Canada and the US, while notably, most respondents in Japan and Mexico considered beef either mostly safe or neither safe nor unsafe. The product type was an important variable in safety perception, with organ meat considered the least safe across all consumer groups, while steak and roast were considered safest by all respondents. The study further queried participants' past experience with food-borne illnesses, with 18.4% (Japan) to 39.7% (Mexico) of respondents having a family member who had experienced food-borne illness.

Branscheid et al. (71) investigated consumers' sensory ratings in relation to sampled beef and lamb steaks; risk perception, knowledge or information requirements about beef were not measured. Branscheid (72) discusses the quality of beef including the proportion of muscle to fatty tissue in a review paper. Risk perception relating to beef is not discussed.

### Key Themes Extracted From the Literature Review: Risk Perception Relating to Red Meat

#### Informational Engagement

A tendency to avoid information may result in more positive attitudes toward and perceived knowledge about red meat, with forced engagement reducing positive attitudes and increasing perceived knowledge in individuals high and low in information avoidance (74). This would indicate that informational engagement, though not sought out voluntarily by information avoidant individuals, reduces positive attitudes toward red meat overall. This contrasts with other results indicating that individuals less likely to engage heuristic cognitive processing of food choices (higher educational attainments, less influenced by mass media, engaging with food label information) may have higher beef safety perceptions (70), indicating a latent presence of heuristic risk perception primes may boost everyday risk perception in the population (e.g., due to coverage of food scares). The distinct constructs of beef safety perception and attitudes toward red meat may be differently affected by informational engagement and availability.

Information sources relied on to inform consumption behaviour vary between individuals are likely to vary across cultures. Many Polish consumers (*N* = 1,004) reported being influenced by own expertise, medical advice and opinions of others such as family and friends while other sources, such as press articles, advertising, labelling information, seller's recommendations, or exposition in the store were rated as little influential (75). Similarly, Hornibrook et al. (76) find that consumer-dominated sources of information are rated as most useful by consumers, indicating the importance of personal experience for information sources to be considered trustworthy.

#### Risk and Health Perceptions and Trust in Production Safety Standards and Regulation/Food Safety Sector

Fears regarding beef safety, linked to confidence in production standards and regulation, are one of the key barriers to meat consumption. Such fears are linked to different information engagement strategies, with the types of information sources that are trusted and the readiness to engage with risk or health information affecting individuals' risk and safety perceptions.

Positive health associations with beef consumption were found in Polish consumers, particularly in younger age groups (75); participants from Germany, Spain, France, and the United Kingdom [(79); eight focus groups, *N* = 65] also predominantly reported associations with positive health effects and nutritious value.

There are some indications of low awareness of negative health impacts of beef with respect to carcinogenic and cardiovascular disorders from small samples across European nations (79), with some larger studies (80) indicating an overall confidence in the safety of beef products on the market, although a population representative survey of Spanish consumers (70) found low beef and moderate pork safety perceptions. Health risks may be perceived to depend on consumption factors such as amount, type, preparation method, and presence of harmful residues (79); variations in risk perception could therefore be a result of different assumptions made by participants regarding consumption factors. Given the link between beef safety perception, media reporting, and trust in production standards and food safety regulation, there are likely to be fluctuations in safety perceptions over time and across nations.

## Discussion

### NovRBA Communication Strategy

The findings summarised above were synthesized into a framework for a communication strategy for the NovRBA project's results. The key challenge for communicating the project's results was finding a way to communicate risks and benefits of a food choice (specifically, red meat or insects) jointly. As little of the literature reviewed above investigates the simultaneous communication of risks and benefits, the strategy was derived on the basis of expectations of how such risk-benefit communication would likely interact with the cognitive and emotional processes affecting insect and meat consumption choices identified from the literature reviews. The principles outlined below constitute the framework for the NovRBA communication strategy which will guide how final project results are communicated.

Different applications of this framework are possible, depending on whether the project results indicate an overall health benefit of diets replacing red meat with insects or vice versa, or no overall health advantage of either type of diet. Across these three possible outcome scenarios, the core challenge for communication is navigating the relative benefits of either type of diet, as the comparison between them has inherent implications for the audience's interpretation. Outlined below are some of the specific aspects which need to be considered for communication choices in order for communication not to elicit emotional responses which could affect behaviour in an unintended way directly opposed to the informational content communicated. Communication choices and settings would be differentially favourable or unfavourable for each type of diet; consequently, the balance between different options (guided by the principles below) must be determined by the NovRBA project outcomes, specifically, by which type of diet (if any) is found to have the more positive overall health impact.

If aiming to promote insect consumption and health perception, any risk-benefit communication relating to insect consumption must avoid triggering animal reminder disgust or increasing risk perception by evoking associations with live animals (either through visual or text information). Consequently, animal-shaped visual imagery (including abstract imagery such as found in logos) should be avoided. Text descriptions should be emotionally neutral and avoid animal references. However, given the importance of trust in informational sources when weighting information received, it is imperative to maintain transparency on the insect content of food.

Given the non-commercial incentives of this study and its associated communication, a collective rather than individual framing of health benefits may yield better results. Specifically, highlighting consumption health benefits to vulnerable groups (e.g., individuals at risk from meat consumption, individuals suffering from nutrient deficiency) and potential indirect benefits of increasing perceived social acceptability of insect consumption by normalising consumption behaviour (e.g., by reducing barriers to consumption, such as perceived social stigma, for vulnerable groups) may result in stable, long-term, positive effects on consumption behaviour.

Opportunities for consumers to engage in-depth with information about the relative risks and benefits of insect consumption in a trusted setting seem likely to facilitate information-based behavioural engagement and attitudes. Settings likely to facilitate engagement in the context of reduced barriers could be information or tasting events offered by trusted organisations (these may vary culturally, but may include scientists, government and consumer protection organisations rather than organisations or individuals with commercial interests). The role of independent public organisations such as the German Federal Institute for Risk Assessment is usually not considered in research in this field due to their specialised nature as well as the challenges of different structures of consumer protection across different countries. However, due to their combination of scientific approaches, impact on the regulatory sector for food safety, and independence from the food industry, it could be hypothesized that they would be particularly well-placed to provide information or engagement opportunities to individuals aware of the organisations' roles and responsibilities.

A social setting (e.g., family events or friend groups) would be likely to promote positive engagement with both risk and benefit considerations; the opportunity to discuss concerns and benefits with trusted individuals (this could include scientific researchers present) but also with close connections whom concerns are likely more easily shared with and whose judgements and thought processes are well-known and trusted (i.e., family, partners, or close friends) may provide social support reducing emotional barriers such as animal reminder disgust and thus facilitate information-based engagement. The likely boosts of such a social setting to social norm perceptions may increase the likelihood of consumption behaviour, though any such effects would depend on the specific social group, as disgust reactions of any individuals within the group may negatively affect other group members. Contexts characterised by positive exploratory (novelty-seeking) behaviour and willingness to engage with scientific information in a playful manner, such as science-based fairs, outreach or university events, may promote positive engagement, foster positive experiences, and thus indirectly improve social acceptability of entomophagy in Western cultures.

While product safety and consumption risks play a large role in beef consumption and perceptions, this factor seems to contribute to a far lesser degree to insect consumption decisions in Western cultures. This is likely a result of edible insects being less established as a food source; consequently, emotional barriers such as food neophobia play a disproportionately larger role. Additionally, the more established meat industry has experienced past food crises, which have impacted public opinion and, in some cases, eroded trust in product safety. Because insects are not consumed at a large scale in Western cultures, it is possible that individuals, in the absence of food scares prompting considerations about consumption risks, assume that regulatory frameworks and consumer protection mechanisms ensure that products available for purchase are safe to consume. Consequently, providing transparent information about food safety may impact consumers' decisions on insect consumption less than on meat consumption. A similar mechanism may apply to information about consumption health risks and benefits: emotional barriers may prevent engagement with informational considerations regarding consumption choices. Therefore, providing settings which allow emotional barriers to be overcome (see above) is likely to be crucial to allow individuals to engage with health risk and benefit information and make information-based consumption choices.

Associations of red meat with both positive and negative health outcomes found in various studies indicate a degree of awareness among the European population about both risks and benefits of red meat consumption. Establishing informational engagement with trusted sources of information is key to supporting informed consumption choices reflecting risks as well as benefits. Given that red meat is well-established on European markets, cultural aspects (such as social acceptance) or emotional barriers to consumption play a reduced role compared to insect consumption choices. Consequently, a comprehensive, neutral, joint communication of risks and benefits of red meat consumption from trusted information sources is likely to succeed in promoting informed consumption choices. Given the long-term nature of some health risks and benefits to be communicated, one of the most trusted informational sources, consumer-to-consumer exchange of past experiences, is unlikely to be useful for raising awareness as long-term benefits are not observed immediately and will thus not be included when relating personal product experience. However, consumer organisations may be in a position to promote informed consumption choices by delivering neutral information about long-term health risks and benefits as an information source comparatively little removed from consumers' firsthand experience.

### Challenges and Limitations

The limited scope of the literature review was a challenge for the design of the communication strategy. The same search terms (relating to risk perception and acceptance) were applied in the literature search regarding red meat and that on edible insects. However, due to the novelty of edible insects on Western markets compared with red meat, it is likely that more emotional and cognitive factors other than risk perception (e.g., disgust) affect consumption behaviour. The search was widened to include acceptance to accommodate for this; however, it is likely that acceptance affected meat consumption far less than insect consumption. The search terms were thus a compromise between comparability between the two literature searches and a search tailored to the specific field; however, it is likely that this resulted in the exclusion of relevant publications that could have been identified using a more inclusive search strategy tailored more specifically to each of the fields in question.

One of the key challenges in developing an evidence-based communication strategy on the basis of the literature reviews described was the lack of standardisation and comparability across samples, methodologies, chosen variables, and reporting standards in the literature reviewed. For example, because few studies collected data from samples representative of national populations, little could be inferred about the impact of cultural differences, which ought to be considered (14). The statistical data reported in the reviewed articles differed greatly, with some studies not reporting any relevant numerical or descriptive data, some reporting means but not standard deviations, and some providing no or incomplete descriptive statistics for the sociodemographic criteria of the samples studied. This arises in part from the range of methodologies and statistical techniques applied, as well as the different standards for statistical reporting in different fields and disciplines.

It was overall challenging to synthesize the available information into a coherent picture of consumers' acceptance and risk perception relating to edible insects compared to red meat. Studies, especially on acceptability of insect-based foods, report directly opposing findings in some instances. It is impossible to differentiate whether such cases reflect cultural or sociodemographic differences between samples, arise from methodological differences, or represent statistical artefacts. Furthermore, due to the general lack of reporting for effect sizes, comparisons of different findings cannot easily be quantified. Nevertheless, several key trends in consumer acceptance of insect-based foods could be identified based on the literature reviewed above. Some findings indicated that behavioural intention (e.g., self-reported willingness to consume insects) differed from actual behaviours (e.g., insect consumption). This poses a challenge for any inferences made from studies using proxy measures for behaviour, such as behavioural intention, for the design of the communication strategy. However, it is likely that behavioural measures are less sensitive than behavioural intention or attitude measures; an improvement in attitude, for example, may not be sufficient to cause a behavioural change, but may constitute an incremental contribution toward more favourable behavioural intentions, which may in turn require incremental increases before a behavioural change is observed. Consequently, study designs relying on such proxy measures for behaviour were still considered for the communication strategy design.

Investigating health and risk perceptions regarding beef consumption, similar issues were encountered. For example, some large-scale studies found contradictory results indicating the Spanish population perceived beef products as unsafe compared to other products (70) or were confident about beef products (80). Such findings are likely either a result of methodological variations or changes in perception over time and were particularly challenging to interpret for the design of the communication strategy. Similarly, some sociodemographic differences, such as age effects on beef health perceptions (75) or the relative rating of past experience as useful information (76) and education differences informational engagement (70) were observed; however, due to methodological variations and confounding cultural differences, no trends could be identified from the literature reviewed. Though challenging with respect to the communication strategy, these findings highlight that beef risk and health perceptions are not resistant to information received from external sources. Current inconsistencies may arise from conflicting information received from different sources as well as cultural variations in traditional views of beef consumptions. This highlights the importance of further advancing our understanding of consumption health risks and benefits and their consistent and transparent communication, as these information may impact consumer perceptions and thus be vital in promoting healthy consumption choices.

This study highlights the challenges of designing a communication strategy for the health risks and benefits associated with consuming a novel compared to an established food. It is crucial to consider not only the scientific information to be communicated, but the audience's informational and emotional needs for communication to promote healthy, informed consumption decision-making. For insect and meat consumption, the high contrast between the factors impacting their respective consumption choices presented a particular challenge. Communicating health impacts of other red meat replacers may involve less complex considerations; for example, pulses or laboratory-grown meat-alternatives may not evoke disgust reactions. This study highlights the importance of considering the audience's needs carefully, both in terms of information basis and emotional factors impacting consumption considerations and choices. A one-size-fits-all approach to food health communication would have been unlikely to promote healthy consumption choices in the case at hand. A communication approach tailored to the audience's needs may enable consumers to make informed consumption choices.

## Conclusion

This paper presents how a framework was generated based on two literature reviews in order to communicate the results of a risk-benefit assessment of the health impacts of replacing red meat with edible insects. For edible insects, disgust and animal reminder, food neophobia and familiarity, state of food (processed vs. unprocessed), contextual information, cultural differences, social norms and contexts, and sociodemographic characteristics, were identified as likely to affect consumption choices. Red meat consumption choices were considered impacted by informational engagement and risk and health perceptions and trust in production safety standards and regulation/food safety sector. Communicating health risk-benefit trade-offs of foods and comparing two types of food is a complex challenge, in particular when considering novel foods because little data is available about consumers' informational needs or emotional barriers or consumption facilitators. Yet, these are vital to consider before communication choices. Otherwise, communication aiming to support and promote healthy consumer choices may unintentionally evoke emotional responses (such as disgust elicited through pictures of insects that trigger animal reminder reactions) which prevent information-based decision-making about healthy consumption choices. Communication choices about risk-benefit assessments should therefore be based on the information available about consumers' emotional barriers or facilitators as well as their informational needs where possible.

## Data Availability Statement

The original contributions presented in the study are included in the article/supplementary material, further inquiries can be directed to the corresponding author/s.

## Author Contributions

The study was designed in the context of the NovRBA project, with contributions from EB, ML, G-FB, and DB to the design and method. DB conducted the literature search. EB and DB screened the identified literature and extracted information from the eligible articles. EB, DB, and EV interpreted the information and wrote the manuscript. The ongoing work on the communication strategy regarding the NovRBA project's findings was supervised by ML and G-FB. All authors provided input to different versions of the manuscript.

## Funding

This publication is based on the NovRBA project (grant number: GP/EFSA/ENCO/2018/03-GA01), funded by EFSA.

## Author Disclaimer

EV is employed with EFSA in the Nutrition Unit. However, the present article is published under the sole responsibility of the author and may not be considered as an EFSA scientific output. The positions and opinions presented in this article are those of the author alone and do not necessarily represent the views or scientific work of EFSA. To learn about the views or scientific outputs of EFSA, please consult its website under http://www.efsa.europa.eu. The NovRBA project launched in March 2019 under the support of EFSA [grant number GP/EFSA/GP/EFSA/ENCO/2018/03-GA01] and is coordinated by the National and Kapodistrian University of Athens (NKUA). Sole responsibility lies with the authors and the Authority is not responsible for any use that may be made of the information contained therein.

## Conflict of Interest

The authors declare that the research was conducted in the absence of any commercial or financial relationships that could be construed as a potential conflict of interest. The reviewer CP declared a shared affiliation with one of the authors EV to the handling editor at the time of review.

## Publisher's Note

All claims expressed in this article are solely those of the authors and do not necessarily represent those of their affiliated organizations, or those of the publisher, the editors and the reviewers. Any product that may be evaluated in this article, or claim that may be made by its manufacturer, is not guaranteed or endorsed by the publisher.
